# The Effect of Local Injection of Epinephrine and Bupivacaine on Post-Tonsillectomy Pain and Bleeding

**Published:** 2013-10

**Authors:** Ali Reza Bameshki, Marzieh Razban, Ehsan Khadivi, Majid Razavi, Mehdi Bakhshaee

**Affiliations:** 1 *Cardiac Anesthesia Research Center, Imam Reza Hospital, Faculty of Medicine, Mashhad University of Medical Sciences, Mashhad, Iran.*; 2 *Department of Otorhinolaryngolog, Imam Reza Hospital, Mashhad University of Medical Sciences, Mashhad, Iran.*; 3 *Sinus and Surgical Endoscopic Research Center, Imam Reza Hospital, Faculty of Medicine, Mashhad University of Medical Sciences, Mashhad, Iran.*

**Keywords:** Bleeding, Bupivacaine, Epinephrine, Post-tonsillectomy pain, Post-tonsillectomy, Tonsillectomy

## Abstract

**Introduction:**

Tonsillectomy is one of the most common surgeries in the world and the most common problem is post-tonsillectomy pain and bleeding. The relief of postoperative pain helps increase early food intake and prevent secondary dehydration. One method for relieving pain is peritonsillar injection of epinephrine along with an anesthetic, which has been shown to produce variable results in previous studies. Study Deign: Prospective case-control study. Setting: A tertiary referral centers with accredited otorhinolaryngology-head & neck surgery and anesthesiology department.

**Materials and Methods:**

Patients under 15 years old, who were tonsillectomy candidates, were assigned into one of three groups: placebo injection, drug injection before tonsillectomy, and drug injection after tonsillectomy. The amount of bleeding, intensity of pain, and time of first post-operative food intake were evaluated during the first 18 hours post operation.

**Results:**

The intensity of pain in the first 30 minutes after the operation was lower in the patients who received injections, but the difference was not significant during the first 18 hours. The intensity of pain on swallowing during the first 6 hours was also lower in the intervention groups as compared with the placebo group. The amount of bleeding during the first 30 minutes post operation was lower in the two groups who received injections, but after 30 minutes there was no difference.

**Conclusion:**

Injection of epinephrine and bupivacaine pre- or post- tonsillectomy is effective in reducing pain and bleeding. The treatment also decreases swallowing pain in the hours immediately after surgery.

## Introduction

Tonsillectomy is one of the most common surgeries in the world. Despite advancements in surgical and anesthetic techniques, bleeding and pain are still the most common complications of tonsillectomy (1). Since pain relief after tonsillectomy can help patients recommence food intake and can prevent patient dehydration secondary to low food intake, many efforts have been made to find an effective approach. Several different treatment techniques have been developed for use during and after surgery, including steroids, analgesics, antibiotics, and anti-nausea medications, and have been shown to have some positive outcomes in randomized trials. One of these techniques is injection of epinephrine and bupivacaine into the peritonsillar region, which has been reported to have variable outcomes in previous studies (2). 

Considering the inconsistent results of previous studies and the low cost of this technique, we evaluated the effectiveness of peritonsillar injection of epinephrine and bupivacaine on decreasing pain and bleeding after tonsillectomy. 

## Materials and Methods

Patients who were referred to Imam Reza Hospital for tonsillectomy and were under 15 years old were eligible for this double-blind clinical trial. Patients with a history of previous disease or coagulation problems were excluded. After obtaining written informed consent from the patients’ parents, study subjects were assigned into one of three groups: one control group who received placebo injections of saline and two case groups who received injections of medication either pre- or post-operation. The required study sample was calculated to be 11 patients for each group, but for increased precision and to account for possible subject loss, we enrolled 24 patients in eachgroup. Patients in the control group received 4 ml normal saline and patients in the case groups received 4 ml of a solution of bupivacaine (0.5%) and epinephrine (1/200000 concentration). The 4 ml of either saline or drug solution was divided into 2 doses and one dose was injected into each of the upper and lower regions of the tonsils. The surgeon, recovery technician and ward nurses were blinded to the medication type and only completed a study questionnaire based on the patient’s condition. The same surgeon and anesthesiologist performed all the surgeries and the method of anesthesia, dosage of injected medication, and method of surgery were similar for all the subjects. Patient information collected included age, gender, duration of surgery, and amount of bleeding during surgery. The amount of bleeding was estimated based on the number of cauterization, bloody sponges that were collected and the surgeon’s satisfaction with surgical field quality was also graded in a 5-item Likert scale (3). The patient’s condition post-operation was also documented using a questionnaire to report the intensity of pain based on Wong-Baker FACES Pain Rating Scale (4), the time of starting food intake, sleep condition in the first night and the presence of nausea, vomiting, earache, or pain during swallowing in the first 18 hours post-operation. Data were analyzed using the Chi-squared test and Kruskal-Wallis test; P<0.05 was considered statistically significant. 

This study was approved by the Ethics Committee of Mashhad University of Medical Sciences, all parents were fully informed about the study protocol and a signed informed consent was obtained from each of them.

## Results

The distribution of the age and gender of the subjects was similar between the three groups (Table.1). The mean length of surgery for all the groups was similar at 21 minutes and 40 seconds (SD=5.14 min). The amount of bleeding based on the surgeon’s opinion was not significantly different between the groups, but the mean number of cauterization was used in the group who received pre-operative injections was significantly less than in the other two groups (P=0.002). The mean number of sponges used during surgery in the control group and group who received post-operative injections (4.2 and 3.4 sponges, respectively) was more than in the group who received pre-operative injections (2.2 sponges) but the difference was not significant. The amount of bleeding in the first 30 minutes after surgery in the recovery room, was greater in the control group compared with the two intervention groups (P=0.01), but the difference between the two intervention groups was not significant (P>0.05). The amount of bleeding during the 18 hours following surgery was not significantly different between the three groups. 

**Table 1:** Demographic characteristics of the patients among different groups

	Number	Age(Y)±SD	Significance	F/M	Significance
Control Group	24	8.95 ± 3.49	NS	13/11	NS
Pre-Operation Group	24	8 ± 3.31	NS	12/12	NS
Post-Operation Group	24	9.87 ± 2.41	NS	13/11	NS

The intensity of pain based on the FPRS in the 30 minutes after entering the recovery room was higher in the control group compared with the two other groups (P=0.02) but the difference between the two intervention groups was not significant (Table. 2). 

**Table 2:** Pain scaling according to faces pain rating scale (FPRS) 30 minutes post-operation among different group

	**Pain Complaining** **(Number)**	**Pain None-complaining** **(Number)**
Control Group	22	2
Pre-Operation Group	11	13
Post-Operation Group	12	12

The intensity of pain during the remaining post-operative hours was not significantly different between the groups. Swallowing pain at 2, 4, and 6 hours post-operation was less in the intervention groups compared with the control group (P<0.05) but there was no statistically significant difference between the two intervention groups (P>0.05) (Table. 3). 

**Table 3:** Swallowing pain frequency 6 hours after operation among groups

	**Pain Complaining** **(Number)**	**Pain None-complaining** **(Number)**
Control Group	22	2
Pre-Operation Group	11	13
Post-Operation Group	12	12

In the remaining hours after surgery, the intensity of swallowing pain was similar for all the groups (P>0.05). The intensity of earache and the time of starting oral intake of food/liquid did not show any statistically significant differences. The amount of post-operative analgesic used was similar between the groups and was 250 ml of acetaminophen syrup. The sleep condition during first post-operation night has been shown in (Table. 4).

**Table 4:** Sleep condition among groups during post-operation first night

	**Well Sleeping**	**Occasional awaking periods**	**Frequent waking periods**	**No Sleeping**
Control Group		11	10	3	0
Pre-Operation Group		15	7	2	0
Post-Operation Group		16	7	0	1

## Discussion

The most common complication of tonsillectomy, which happens after almost all surgeries, is pain that lasts for approximately one week (5). Therefore we evaluated patients’ pain according to several different direct and indirect variables; post-operative pain based on FPRS, earache, swallowing pain, sleep condition, time of restarting oral intake of food/fluid, and amount of analgesic used during the first 18 hours post-operation. Based on the study results, only the intensity of the patients’ pain during the first 18 hours after surgery was significantly different between the control and intervention groups. It is important to mention that in the evaluation of patients’ post-operative pain and pain after entering the hospital ward, more than half of patients in the intervention groups reported pain scores of 2 (‘hurts little more’) or 3 (‘hurts even more’) on a scale of 0 to 5; scores which are indicative of moderate pain. In the control group, one third of the patients reported pain scores of 2 or 3, and one third of the patients reported a pain score of 5, indicative of the worst possible discomfort. However, these differences were not statistically significant. In the first 6 hours after surgery, almost half of the patients who received injections did not have any swallowing pain, while all the patients in control group had swallowing pain. It can be interpreted from the results that injection of bupivacaine and epinephrine into the peritonsillar area pre- or post-operation decreases patients’ pain in recovery, and decreases swallowing pain in the first 6 hours after surgery, but does not have any effects on reducing long-term pain. These results contradict the findings of a study by Strub et al. in 1996, which showed that local injection of bupivacaine pre- or post-operation reduces patients’ pain during the first 24 hours after surgery (6). Other studies have reported that injection of local anesthetic does not have any effects on reducing post-operative pain (7,8). Most of the studies that used local infiltration of anesthetics in the peritonsillar area reported pain relief only immediately post-operation^4^, which is similar to the pain relief during the first 30 minutes post-operation, observed in our study. Only a few studies have investigated pain relief over 5 days post-operation, such as the study by Park et al. in 2001 where ropivacaine was administered with or without clonidine. The results of the study indicated that the treatment resulted in pain relief at Days 0, 3 and 5 after surgery (9), and the incidence of earache was reported to be less in the two intervention groups compared with the control group. However, in our study the incidence of earache during the 18 hours immediately after surgery was similar between the three groups.

In this study we did not find any significant differences in pain relief between the two intervention groups (those who received injections pre- or post-operation) but the amount of bleeding was different between the groups. Similarly, in another double-blind study on 80 adults who underwent tonsillectomy, no differences were reported in pain relief between two patient groups who received injections of ropivacaine pre- or post-operation. However, a reduction in immediate postoperative pain was reported in the two groups who received the injection compared with the control group (10). Regarding bleeding, from the results of this study it can be interpreted that bleeding during surgery in the group who received pre-operative injections was less than in the control group, although only one of the tested variables showed a significant difference. In evaluating bleeding at different time points post-operation, only the amount of bleeding in the first 30 minutes in the recovery room (post-operative immediate bleeding) was significantly different between the intervention groups and the control group (greater in the control group). Injection of anesthetic alone does not have an effect on postoperative bleeding (11), but according to most previous studies, injection of epinephrine with an anesthetic decreases bleeding during the operation but a reduction in postoperative bleeding is not mentioned (12). However, in the present study, a reduction in bleeding immediately after surgery (within the first 30 minutes) was observed, which could be explained by the short duration of effect of epinephrine. Administrating bupivacaine along with epinephrine more efficiently reduces bleeding and increases the pain relief period (13).

## Conclusion

Local injection of epinephrine with bupivacaine in the peritonsillar area before tonsillectomy can decrease bleeding during surgery and administration of the drugs either pre- or post-operation reduces immediate bleeding. Pre- or post-operative injection of epinephrine with bupivacaine also reduces immediate post-operative pain, and decreases swallowing pain in the hours immediately after surgery; therefore their use will help to keep patients calm and recommence oral food intake as soon as possible.
